# Supplementary material for: The transcription factor odd-paired regulates temporal identity in transit-amplifying neural progenitors via an incoherent feed-forward loop
Source: eLife. 2019 Jul 22;8:e46566. doi: 10.7554/eLife.46566 (PMC6645715; doi:10.7554/eLife.46566)
Supplement: Supplementary file 1. [file elife-46566-supp1.docx]

**Figure 1-supplementary Table 1**

| **gene_id** | **current_symbol** | **log2FC Grh vs D** | **log2FC Grh vs Ey** | **D^+^ INPs** | **Grh^+^ INPs** | **Ey^+^ INPs** |
| --- | --- | --- | --- | --- | --- | --- |
| FBgn0263511 | Vsx1 | -2.38823 | -0.966157 | 52.8282 | 9.7357 | 16.34 |
| FBgn0003300 | run | -1.37337 | -1.112051 | 298.302 | 121.75 | 221.5 |
| FBgn0034436 | CG11961 | -1.27932 | -0.641504 | 139.823 | 61.576 | 80.37 |
| FBgn0052016 | 4E-T | -1.19884 | -1.565084 | 261.48 | 96.775 | 287.7 |
| FBgn0003002 | opa | -1.02134 | -0.861077 | 589.571 | 309.76 | 469.3 |
| FBgn0040232 | cmet | -1.01771 | -1.288723 | 262.961 | 93.662 | 273.3 |
| FBgn0020503 | CLIP-190 | -0.97462 | -1.431886 | 142.998 | 65.083 | 169.4 |
| FBgn0040233 | cana | -0.96022 | -1.268322 | 90.4868 | 44.278 | 95.48 |
| FBgn0050007 | CG30007 | -0.95713 | -1.259524 | 85.0007 | 40.344 | 89.17 |
| FBgn0259994 | CG42492 | -0.8325 | -1.268517 | 108.422 | 62.722 | 126.3 |
| FBgn0039936 | Gyf | -0.79879 | -1.201766 | 390.699 | 218.78 | 447.9 |
| FBgn0262117 | IntS3 | -0.77855 | -1.144134 | 359.032 | 204.4 | 400.4 |
| FBgn0010247 | Parp | -0.77277 | -1.194843 | 186.475 | 87.538 | 225.9 |
| FBgn0032223 | GATAd | -0.76409 | -1.157956 | 73.7891 | 43.322 | 84.12 |
| FBgn0051510 | CG31510 | -0.76124 | -1.689937 | 73.6238 | 41.551 | 130.1 |
| FBgn0262743 | Fs(2)Ket | -0.739 | -1.255325 | 1003.28 | 592.68 | 1251 |
| FBgn0036309 | Hip1 | -0.70421 | -1.249384 | 92.4375 | 44.882 | 129.3 |
| FBgn0052000 | CG32000 | -0.68624 | -1.418639 | 351.246 | 211.82 | 525.5 |
| FBgn0013343 | Syx1A | -0.66268 | -1.47396 | 61.5255 | 30.552 | 113.2 |
| FBgn0024728 | Slip1 | -0.66241 | -1.492769 | 207.484 | 131.5 | 339.5 |
| FBgn0025740 | PlexB | -0.65846 | -1.103509 | 96.1694 | 51.242 | 119.4 |
| FBgn0022361 | Pur-alpha | -0.64159 | -1.581099 | 152.94 | 95.057 | 280.6 |
| FBgn0025741 | PlexA | -0.63588 | -1.229105 | 374.736 | 198.81 | 544.2 |
| FBgn0046706 | Haspin | -0.63494 | -1.096967 | 149.075 | 80.767 | 189.3 |
| FBgn0265297 | pAbp | -0.62838 | -1.851062 | 724.31 | 463.77 | 1594 |
| FBgn0026379 | Pten | -0.62607 | -1.302109 | 102.635 | 64.62 | 150.1 |
| FBgn0035617 | l(3)psg2 | -0.62177 | -1.149762 | 81.3586 | 51.266 | 103.7 |
| FBgn0266937 | CR45388 | -0.61669 | -1.274405 | 70.7628 | 32.981 | 115.8 |
| FBgn0039928 | Cals | -0.6117 | -1.544939 | 237.117 | 123.9 | 498.5 |
| FBgn0023213 | eIF4G | -0.60592 | -1.211427 | 641.831 | 360.26 | 942.7 |
| FBgn0026401 | Nipped-B | -0.59981 | -1.509991 | 260.928 | 120.5 | 571.5 |
| FBgn0026262 | bip2 | -0.59465 | -1.621588 | 105.174 | 50.163 | 258.8 |
| FBgn0261934 | dikar | -0.58721 | -1.222149 | 214.456 | 142.17 | 296.8 |
| FBgn0053554 | Nipped-A | -0.58326 | -1.528434 | 468.037 | 233.2 | 1029 |
| FBgn0052479 | Usp10 | -0.58049 | -1.33511 | 234.942 | 156.87 | 361.3 |
| FBgn0003165 | pum | -0.56773 | -1.232784 | 259.928 | 175.98 | 371.5 |
| FBgn0025936 | Eph | -0.56652 | -1.319311 | 352.417 | 237.69 | 540.6 |
| FBgn0040011 | Slmap | -0.56309 | -1.143017 | 356.135 | 246.74 | 479.8 |
| FBgn0014388 | sty | -0.55656 | -1.235945 | 58.1928 | 39.015 | 83.17 |
| FBgn0264491 | how | -0.55544 | -1.661452 | 52.9546 | 34.659 | 113.3 |
| FBgn0262972 | CR43283 | -0.55318 | -1.288425 | 17783.6 | 6996.6 | 35362 |
| FBgn0027101 | Dyrk3 | -0.542 | -1.282217 | 163.438 | 111.34 | 248.9 |
| FBgn0026869 | Thd1 | -0.53965 | -1.447248 | 400.909 | 270.71 | 712.7 |
| FBgn0261574 | kug | -0.53274 | -1.28185 | 122.889 | 83.634 | 184.2 |
| FBgn0037025 | Spc105R | -0.52595 | -1.146529 | 226.093 | 160.85 | 313 |
| FBgn0052296 | Mrtf | -0.5145 | -1.009835 | 61.6226 | 43.605 | 78.54 |
| FBgn0000541 | E(bx) | -0.51104 | -1.147588 | 656.885 | 413.94 | 995.5 |
| FBgn0262124 | uex | -0.50195 | -1.146037 | 178.679 | 127.05 | 248.6 |
| FBgn0039920 | CG11360 | -0.50189 | -1.490281 | 293.349 | 206.09 | 549.9 |
