# Supplementary material for: The transcription factor odd-paired regulates temporal identity in transit-amplifying neural progenitors via an incoherent feed-forward loop
Source: eLife. 2019 Jul 22;8:e46566. doi: 10.7554/eLife.46566 (PMC6645715; doi:10.7554/eLife.46566)
Supplement: Supplementary file 2. [file elife-46566-supp2.docx]

**Figure 1-supplementary Table 2**

| **gene_id** | **current_symbol** | **log2FC Grh vs D** | **log2FC Grh vs Ey** | **D^+^ INPs** | **Grh^+^ INPs** | **Ey^+^ INPs** |
| --- | --- | --- | --- | --- | --- | --- |
| FBgn0003651 | svp | 6.0045875 | 0.710535153 | 1.4014 | 163.1 | 75.1168 |
| FBgn0039678 | Obp99a | 2.698182474 | 0.987302876 | 6.306 | 53.18 | 21.6179 |
| FBgn0261953 | TfAP-2 | 2.448475728 | 0.902912394 | 121.87 | 769.1 | 328.988 |
| FBgn0036786 | skl | 2.3694497 | 1.333899787 | 9.6159 | 74.08 | 20.2187 |
| FBgn0283508 | nw | 2.322193504 | 2.520788487 | 13.318 | 87.71 | 10.0829 |
| FBgn0263240 | Coop | 2.308510822 | 1.326001081 | 8.1282 | 50.41 | 15.9189 |
| FBgn0259834 | out | 1.893328556 | 0.686577009 | 0.3451 | 61.19 | 19.8976 |
| FBgn0033268 | Obp44a | 1.761659329 | 1.077994798 | 112.15 | 488.7 | 181.814 |
| FBgn0261341 | verm | 1.686849862 | 2.341386764 | 13.681 | 51.31 | 7.50242 |
| FBgn0053196 | dpy | 1.68213227 | 1.96520749 | 16.125 | 63.07 | 11.3891 |
| FBgn0259211 | grh | 1.660024793 | 0.669233821 | 165.74 | 663.8 | 306.246 |
| FBgn0038720 | CG6231 | 1.590721124 | 0.863035266 | 24.922 | 86.59 | 39.0646 |
| FBgn0004893 | bowl | 1.551550976 | 1.36873942 | 6.709 | 51.14 | 7.49415 |
| FBgn0016034 | mael | 1.485627173 | 0.674001733 | 18.792 | 62.15 | 32.5101 |
| FBgn0250907 | Cht10 | 1.466894942 | 0.728478866 | 20.702 | 80.01 | 32.6301 |
| FBgn0003731 | Egfr | 1.327910915 | 0.721463379 | 20.432 | 59.49 | 28.0742 |
| FBgn0000557 | Ef1alpha100E | 1.110969319 | 1.442619596 | 37.565 | 92.41 | 27.5523 |
| FBgn0001254 | ImpE2 | 1.109353219 | 2.181882972 | 37.791 | 110.3 | 12.5633 |
| FBgn0085400 | CG34371 | 1.078190182 | 1.007772056 | 29.357 | 78.49 | 26.3762 |
| FBgn0005677 | dac | 1.061218332 | 1.051966667 | 22.809 | 56.95 | 19.3473 |
| FBgn0051030 | CG31030 | 0.84568666 | 1.124481221 | 34.753 | 71 | 26.5327 |
| FBgn0029114 | Tollo | 0.537984034 | 1.032249671 | 41.824 | 68.96 | 25.9713 |
